# Improving image quality and diagnostic usability in photon-counting coronary CT angiography using a novel reconstruction algorithm

**DOI:** 10.1007/s00330-025-11429-z

**Published:** 2025-02-18

**Authors:** Nina P. Haag, Julius H. Niehoff, Iram Shahzadi, Christoph Panknin, Marcus Wiemer, Sven Kaese, Roman Johannes Gertz, Lenhard Pennig, Ole Inuk Platte, Alexey Surov, Jan Borggrefe, Jan Robert Kroeger

**Affiliations:** 1 Department of Radiology, Neuroradiology, and Nuclear Medicine, Johannes Wesling University Hospital, Ruhr University Bochum, Hans-Nolte-Strasse 1, 32429 Minden, Germany; 2 Siemens Healthineers USA, 755 College Road E, Princeton, NJ 08540 USA; 3 Siemens Healthcare GmbH, Henkestraße 127, 91052 Erlangen, Germany; 4 Department of Cardiology, Johannes Wesling University Hospital, Ruhr University Bochum, Hans-Nolte-Strasse 1, 32429 Minden, Germany; 5 Institute for Diagnostic and Interventional Radiology, University Hospital Cologne, Kerpener Str. 62/Gebäude 18a, 50937 Köln, Germany

**Keywords:** Cardiac imaging techniques, Multidetector computed tomography, Image processing, Computer-assisted

## Abstract

**Purpose:**

Qualitative comparison of image quality and diagnostic usability of the recently introduced ZeeFree (ZF) reconstruction algorithm for photon-counting coronary CT angiography (cCTA) with Standard (SD) and TrueStack (TS) reconstruction algorithms.

**Methods and materials:**

This retrospective single-center study included 59 patients (mean age 62.5 ± 13.7, 37 males) who were referred for cCTA on a clinical photon-counting CT scanner between July and December 2023. Curved planar reformations were reconstructed for coronary arteries using ZF, SD, and TS algorithms. Three blinded radiologists individually evaluated image quality on a 5-point Likert scale (5 = excellent; median and interquartile range). Differences were evaluated using Friedman’s test with pair-wise post-hoc testing. Readers were advised to assess the image quality to be diagnostic using a dichotomous yes/no question, with results being presented as percentages and significance evaluated by the Pearson Chi-Square test. Interrater reliability for image quality used Gwet’s AC2 coefficient with ordinal weights, and Gwet’s AC1 coefficient for diagnostic usability.

**Results:**

ZF showed superior quality (4 (2)) compared to SD (4 (2), *p* = 0.03) and TS (4 (1), *p* < 0.001) while a substantial inter-rater agreement was observed for all algorithms (0.70–0.74). Furthermore, ZF obtained the highest diagnostic usability compared to SD (82.5% vs. 77.0%, *p* < 0.001) and TS (75.5%, *p* < 0.001) while yielding an almost perfect inter-rater agreement (0.84).

**Conclusion:**

Compared to SD and TS, the novel ZF image reconstruction algorithm for photon-counting cCTA provides improved image quality and diagnostic usability, suggesting its primary use for clinical assessment.

**Key Points:**

***Question*** *How well does the novel ZeeFree algorithm address motion artifacts in photon-counting coronary CT angiography?*

***Findings*** *ZeeFree significantly outperformed Standard and TrueStack reconstruction algorithms for image quality and diagnostic usability*.

***Clinical relevance*** *Enhanced image quality with ZeeFree could improve the diagnosis of coronary artery disease*.

## Introduction

In non-invasive coronary artery imaging, photon-counting coronary CT angiography (cCTA) has recently emerged as a performant new technology [1]. In the past years, extensive literature has shown its high diagnostic performance, often in comparison with conventional cCTA or invasive coronary angiography [2–6]. Photon-counting CT addresses common challenges associated with cCTA, including motion artifacts, noise reduction, blooming artifacts, or quantification of coronary artery stenosis [7–9]. The depth of these investigations reflects an ongoing effort to refine and optimize photon-counting cCTA for routine clinical use.

In this context, improved spatial resolution with ultra-high-resolution (UHR) cCTA has recently shown its usefulness in patients with severe coronary calcifications, stent imaging [5, 9–11], and plaque imaging, harboring a potential shift in clinical management [12]. The promise of refined and accurate information holds potential implications for improved patient risk stratification, highlighting the potential clinical relevance of this technology. Furthermore, spectral imaging can enhance the performance of photon-counting cCTA by addressing challenges like calcium blooming and metal artifacts through the reconstruction of virtual monoenergetic images [13–16]. These advantages, from optimizing vessel contrasts to potentially reducing contrast agent usage, have the potential to increase diagnostic performance. However, respiratory and cardiac motion artifacts still pose a relevant challenge in photon-counting cCTA, leading to reduced image quality and diagnostic accuracy with subsequently compromised precision of coronary artery assessment [8, 17–19].

Recently, a novel software was introduced for photon-counting cCTA (VB10, syngo.CT, Siemens Healthineers), including a new reconstruction algorithm (ZeeFree (ZF)). This algorithm was specifically developed to reduce respiratory and cardiac motion artifacts in order to potentially enhance image quality and diagnostic utility in cardiac CT [20].

The purpose of this study was to evaluate the assessment of image quality and diagnostic utility of the ZF reconstruction algorithm compared to Standard (SD) and TrueStack (TS) reconstruction algorithms in photon-counting cCTA.

## Materials and methods

### Study design and patient selection

This retrospective study was executed in accordance with the guidelines of the Declaration of Helsinki and received approval from the institutional review board. No financial support from the industry was received. Due to the retrospective nature of the study, the need for patient consent was waived by the institutional review board.

The internal database at a tertiary care medical center was retrospectively reviewed for contrast-enhanced photon-counting cCTA studies performed between July and December 2023. Examinations were included if patients age > 18 years received a standardized protocol cCTA in clinical routine for the assessment of coronary artery disease comprising obligatory image reconstructions of cCTA using ZF, SD, and TS. Examinations conducted in sequential or spiral scan mode were statistically processed, while examinations in high-pitch spiral (flash) mode or repeated exams were excluded from further analysis. Patient characteristics were obtained from patient charts.

### Scan protocol

All scans were performed on a clinical, dual-source photon-counting CT system (NAEOTOM Alpha, software version syngo.CT VB10, Siemens Healthineers). Tube voltage was set at 120 kV with automatic tube current modulation for all scans (Image Quality Level = 100). cCTA was initiated according to a test bolus scan after attenuation exceeded 130 Hounsfield Units in a region of interest placed in the ascending aorta. A total of 50–75 mL of contrast media (350 mg iohexol/mL, Accupaque 350, GE Healthcare) was administered using a dual-syringe power injector at a flow rate of 4 mL/s. The acquisition technique was selected based on the heart rate and rhythm of each patient and was executed in sequential or spiral scan mode. Thresholds were set at 66–80 bpm for sequential mode and > 81 bpm and arrhythmia at any heart rate for spiral mode. If cardiac stents or significant coronary calcifications were present, UHR acquisition mode was used. However, there are currently no official guidelines recommending UHR for specific Agatston scores; clinically, we typically consider an Agatston score of 300 as a reference point. Metoprolol and nitroglycerin were administered for heart rate reduction and vessel dilatation if not contraindicated (up to 15 mg of 1 mg/mL metoprolol tartrate, Beloc® i.v., Recordati Pharma GmbH, and 0.4 mg/pump of nitroglycerin, Glyceroltrinitrat, G. Pohl-Boskamp GmbH & Co. KG).

### Image reconstruction

The ZF, SD, and TS series were primarily reconstructed on the photon-counting CT system using raw CT data. All images were reconstructed in axial orientation using the Bv44 kernel with quantum iterative reconstruction level 3, with either a 512 × 512 image matrix for standard resolution or a 1024 × 1024 image matrix in UHR protocols. Acquisition parameters for the standard-resolution cCTA were 0.4 mm slice thickness with 0.2 mm increment, while spectral UHR cCTA utilized a 0.2 mm slice thickness with 0.2 mm increment. Collimation was 144 × 0.4 mm for standard resolution and 120 × 0.2 mm in UHR protocols.

The SD reconstruction algorithm uses all data available at each image position and blends data in overlapping areas, creating a uniform transition between image stacks. The TS reconstruction algorithm operates by reconstructing each primary axial image from individual image stack data. It partially discards overlapping data, thus producing sharp transitions between images and facilitating the detection of misalignments in coronal or sagittal views [20].

The ZF reconstruction algorithm involves the following key steps: First, a non-rigid registration step is applied independently across all transition zones between image stacks. It selects an image from each stack at the same position within the overlap and measures misalignment by comparing their absolute Hounsfield unit value differences. These images define a 2D plane that maps into a 3D vector field, which is processed using a demon-type registration algorithm to adjust the images for closer alignment. Then, a distance-weighted interpolation and Gaussian smoothing operation is applied across all images and vector fields to reduce tension within the vector fields. The final step is the image reconstruction with user-specific settings (e.g., reconstruction kernel, slice thickness, matrix size, iterative strength, etc.). ZF resembles the base characteristics of TS images by avoiding data mixing in overlap zones, ensuring that each axial image maintains uniform data volume and noise levels, and remaining consistent and immediate contrast enhancement across image transitions at the overlap zones. For a more detailed explanation of the algorithm, we refer to the original publication by Allmendinger et al [20].

### Postprocessing

Postprocessing was performed using a semiautomatic workflow (Coronary workflow, syngo.via, VB80, Siemens Healthineers) and carried out individually for each case on the targeted series, such as ZF, SD, and TS. The coronary anatomy was delineated, and the main coronary arteries were identified and annotated, right coronary artery starting from the ascending aorta, left anterior descending artery including the left main coronary artery, and circumflex branch starting from the bifurcation point of the left main coronary artery. Based on the annotations of the coronary arteries, curved planar reformations were generated and saved as radial ranges. For each case, a total of 9 radial range series were created, representing the three main coronary arteries in the targeted reconstructions ZF, SD, and TS.

### Reader evaluation

Prior to evaluation, each series generated from the radial ranges of the different reconstruction algorithms received a randomly assigned number. Afterward, three readers independently assessed the anonymized radial ranges images: Reader 1, J.R.K., a consultant radiologist with 10 years of experience; Reader 2, J.H.N., a board-certified radiologist with 7 years of experience; and Reader 3, N.P.H., a radiologist in training with 2 years of experience in cardiovascular CT. Anonymized series were presented in random order of case, vessel, and reconstruction. The readers assessed image quality using a 5-point Likert scale, where 1 = Very severe motion artifacts, non-diagnostic, 2 = Severe motion artifacts, significant impact on diagnostic quality, 3 = Moderate motion artifacts, slight impact on diagnostic quality, 4 = Minimal motion artifacts, not affecting diagnostic quality, and 5 = No motion artifacts. Each point on the scale corresponded to the clarity, sharpness, and overall visibility of anatomical structures within the images. The diagnostic usability of each image was assessed using a dichotomous scale, categorized as either diagnostically usable (image quality score ≥ 4) or non-diagnostic (image quality score ≤ 3).

### Statistical analysis

Statistical analysis was performed using SPSS Statistics (version 29.0, IBM), R (version 4.1.0, The R Foundation), and RStudio (version 2023.12.0 + 369, The R Foundation) were used. Ordinal variables are expressed as median and interquartile range or mean and standard deviation. To assess the differences in the distribution of image quality ratings among the three reconstruction algorithms ZF, SD, and TS, the Friedman’s test with pairwise post-hoc testing (Wilcoxon signed-rank test with Bonferroni corrected *p*-values) was employed for the comparative analysis across the three group levels. The interrater reliability for the image quality ratings was evaluated with Gwet’s AC2 coefficient with ordinal weights [21, 22], whereas the interrater reliability for diagnostic usability was assessed using Gwet’s AC1 coefficient with the Pearson Chi-Square test being used to evaluate differences in the evaluation for diagnostic usability. The Gwet’s AC2 coefficients were interpreted as follows: 0.0–0.20 = poor agreement, 0.21–0.40 = fair agreement, 0.41–0.60 = moderate agreement, 0.61–0.80 = substantial agreement, and 0.81–1.00 = almost perfect agreement. The Gwet’s AC1 coefficients were interpreted as follows: values closer to 1 indicating high agreement, and values closer to 0 indicating random agreement. *p*-values less than 0.05 were considered statistically significant.

## Results

### Patient characteristics

**Fig. 1:**
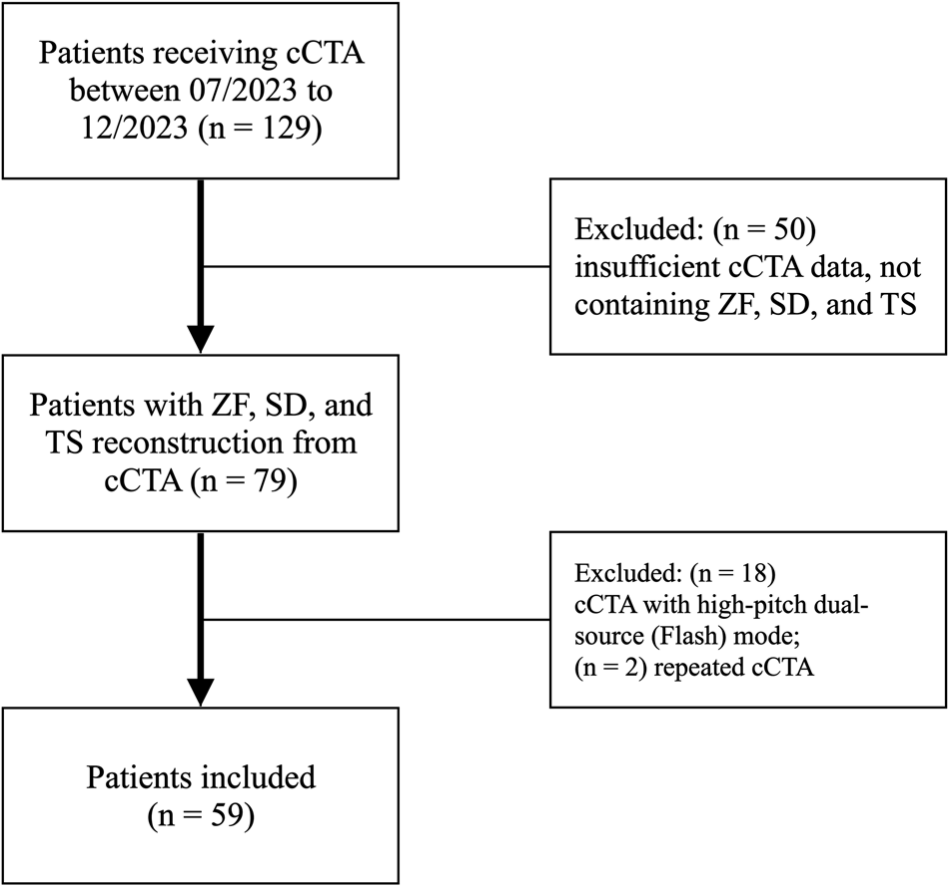
Flow diagram for inclusion and exclusion of patients. cCTA, coronary CT angiography; ZF, ZeeFree reconstruction algorithm; SD, standard reconstruction algorithm; TS, TrueStack reconstruction algorithm; *n*, numbers of patients

One-hundred-and-twenty-nine patients received a cCTA between July and December 2023. Of these, 59 patients (mean age 62.5 ± 13.7 years, 37 males) met the inclusion criteria and were included in the final analysis. Figure 1 illustrates a flow diagram for the inclusion and exclusion of study participants. Table 1 provides a detailed overview of the patient and examination characteristics.

**Table 1:** Demographics and clinical findings of 59 patients

Variable	All patients (*n* = 59)
Age (years)^a^	62.5 ± 13.7
Age range (years)	30–85
Male sex	37 (63)
Female sex	22 (37)
Heart rate (1/min)^a^	68.9 ± 12.1
Arrythmias	9 (15)
Beta blocker usage	57 (97)
Mean dose (mg)^a^	9.2 ± 4.5
Nitroglycerin usage	57 (97)
cCTA acquisition	
Standard-Resolution	11 (19)
Sequential mode	7 (12)
Spiral mode	4 (7)
UHR	48 (81)
Sequential mode	36 (61)
Spiral mode	12 (20)
DLP^a^	410.7 ± 468.6
BMI (kg/m^2^)^a^	29.6 ± 7.0
Agatston Score^b^	78.0 (1270.7)

Data are numbers of patients with percentages in parentheses, unless otherwise noted

*cCTA* coronary CT angiography, *UHR* ultra-high-resolution, *DLP* dose length product, *BMI* body mass index

^a^ Data are means ± SDs

^b^ Data are median and interquartile range

### Image quality

In the study cohort, image quality differed significantly between the three reconstruction algorithms ZF, SD, and TS (*p* < 0.001). After conducting pairwise post-hoc testing, significant differences were observed in image quality ratings between ZF and SD (*p* = 0.030), with ZF exhibiting higher ratings with a median of 4 (2) compared to SD with a median of 4 (2). Similarly, ZF showed better image quality compared to TS (*p* < 0.001), with a median of 4 (1). However, no difference was found between SD and TS (*p* = 0.266).

**Fig. 2:**
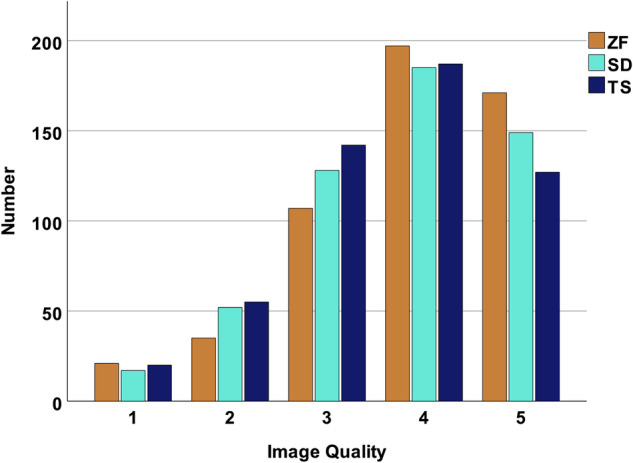
Bar graph displaying the median distribution of image quality ratings on a 5-point-Likert-Scale divided into the different reconstruction algorithms ZeeFree (ZF), Standard (SD), and TrueStack (TS)

Using ZF, 368/531 (69.3%) of all images were rated with a score of 4 and above on the 5-point-Likert scale, whereas the percentages were lower for SD 334/531 (62.9%) and TS 314/531 (59.1%). The distribution of scores is shown in Fig. 2.

The inter-rater agreement analysis using Gwet’s AC2 coefficient with ordinal weights revealed substantial agreement among the three raters in the assessment of image quality: AC2_ZeeFree_ = 0.73 (95% CI: −0.68, 0.78), AC2_Standard_ = 0.74 (95% CI: 0.70, 0.76), and AC2_TrueStack_ = 0.70 (95% CI: −0.66, 0.75).

### Diagnostic usability

**Fig. 3:**
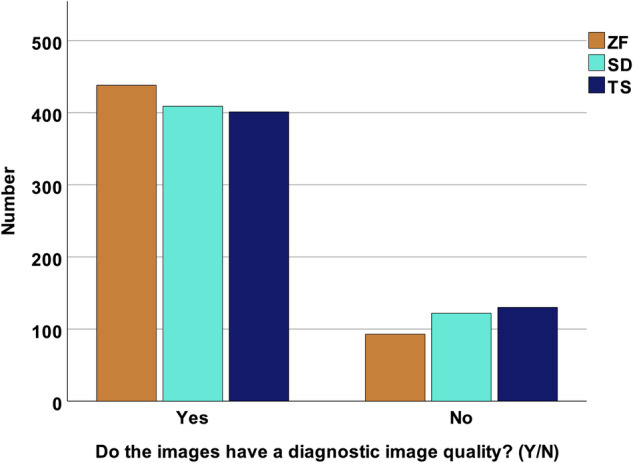
Bar graph displaying the number of images with diagnostic image quality across the different reconstruction algorithms ZeeFree (ZF), Standard (SD), and TrueStack (TS)

ZF images were regarded as diagnostic in 438/531 (82.5%). This was significantly higher compared to SD with 409/531 (77.0%) (*p* < 0.001) and TS with 401/531 (75.5%) (*p* < 0.001). This distribution is visualized in Fig. 3.

The application of Gwet’s AC1 coefficient, accompanied by the Pearson Chi-Square test, demonstrated a good agreement among the three raters in their assessments: AC1_ZeeFree_ = 0.84 (95% CI: 0.78, 0.90), AC1_Standard_ = 0.78 (95% CI: 0.71, 0.86), and AC1_TrueStack_ = 0.68 (95% CI: 0.59, 0.77).

**Fig. 4:**
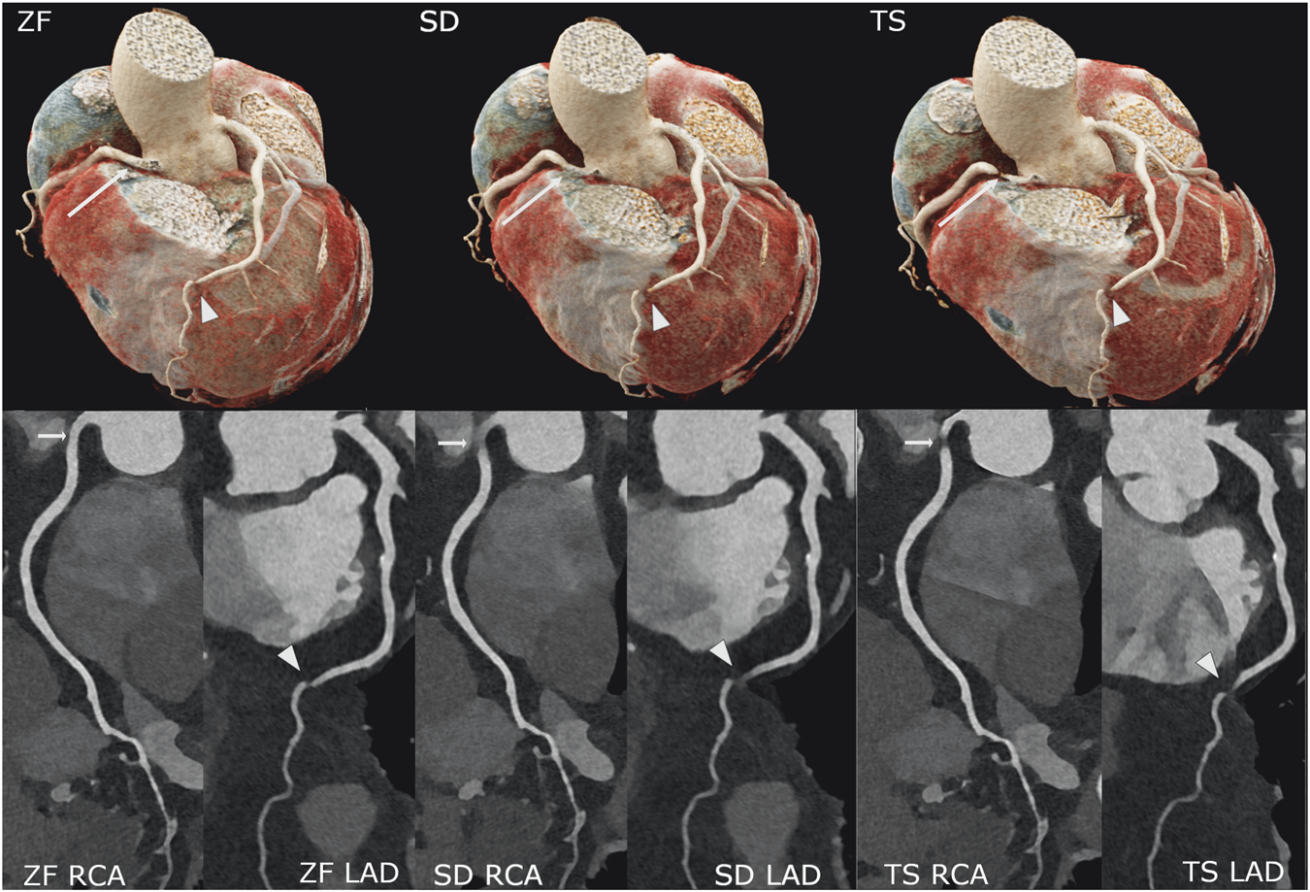
Coronary CT angiography (cCTA) of a 65-year-old female patient was performed for the evaluation of coronary artery disease. The scan was conducted in sequential UHR mode at an average heart rate of 71 bpm, with the administration of 15 mg of beta-blocker and 0.8 mg of nitroglycerin. The upper row displays the 3D heart reconstruction images based on the different reconstruction algorithms: ZeeFree (ZF), Standard (SD), and TrueStack (TS). The bottom row shows curved planar reformations of the right coronary artery (RCA) and the left anterior descending artery (LAD), including the left main coronary artery, for the different reconstruction algorithms. The artifact in the distal LAD (arrowhead) is prominent in TS and SD images but diminished in the ZF image. The arrow indicates a severe artifact in the proximal RCA on TS images, which is reduced in SD images, although the vessel cannot be confidently assessed. In the ZF image, the RCA is displayed in excellent quality without any image artifacts

Figure 4 shows an exemplary cCTA study with the differences in reconstructions and the reduction of artifacts using the ZF algorithm.

## Discussion

This is the investigation of the ZF image reconstruction algorithm in photon-counting cCTA. The qualitative improvements in image quality observed between ZF and both SD and TS reconstructions show the potential of ZF in enhancing the reliability of photon-counting cCTA images. Furthermore, the enhanced diagnostic usability of ZF acknowledges its role in assisting a more effective clinical interpretation.

Artifacts, such as patient-based, physics-based, or scanner-based artifacts, have long been recognized as challenges in CT imaging, especially in cCTA [8]. In terms of patient based artifacts, respiratory and cardiac motion artifacts represent the main concern. In this context, motion correction through patient-based improvements and the application of heart-rate-depended or geometrical image reconstruction algorithms, have shown promising results in enhancing image quality [19, 23]. Building on that, the ZF reconstruction algorithm continues to reduce these artifacts, further improving image quality. This is of particular significance in the context of the diagnosis of coronary artery disease, where accurate and artifact-free images are of utmost interest.

When comparing our study to Moser et al [24] and Lisi et al [25], both emphasize the effectiveness of the ZF reconstruction algorithm in enhancing photon-counting cCTA, yet they focus on different aspects of its application [24, 25]. We evaluated the qualitative image quality of ZF compared to SD and TS algorithms across both sequential and spiral modes in standard resolution and UHR. Moser et al [24] and Lisi et al [25] specifically aimed to evaluate the reduction of stair-step artifacts in sequential UHR cCTA scans only, with the inclusion of CT_FFR_ and plaque analysis. Both studies report that ZF notably enhances the quality of diagnostic images. Additionally, Moser et al find a correlation between the occurrence of artifacts and factors such as heart rate variability and BMI, introducing a layer of patient-specific imaging optimization, while we presented a broader application spectrum of the algorithm across acquisitions modes [24].

The clinical value of this research is underscored by the potential for photon-counting cCTA to evolve into a non-invasive imaging modality, offering detailed assessments of coronary arteries and accurate detection of stenosis and plaque burden [5]. Technical advancements in reconstruction, as provided here by the ZF reconstruction algorithm, not only contribute to enhanced diagnostic usability but also hold the promise of further reducing the necessity for extended and invasive diagnostic procedures. This aligns with the overarching goal of refining and optimizing imaging technologies to streamline diagnostic workflows and improve patient experiences.

Future research should focus on assessing how the improved image quality provided by the ZF algorithm translates into clinical outcomes. This could involve studies that correlate ZF-enhanced imaging with CAD-RADS classification [26], the accuracy of stenosis measurement, and the influence on downstream management decisions, such as the need for further invasive testing or revascularization procedures. Additionally, prospective studies comparing ZF-enhanced imaging with percutaneous coronary angiography could provide information about the correlation to the gold standard, as well as with fractional flow reserve, which could provide insight into the algorithm’s ability to improve non-invasive coronary artery disease evaluation and risk stratification.

However, the study is not without limitations. Its retrospective design introduces inherent biases, including the potential for observer bias, where the interpretation of images may be influenced by the reviewers’ expectations or knowledge of the study’s goals. Varying levels of clinician experience may contribute to variability in exam quality. The reconstruction algorithm is vendor-specific and cannot be generalized to CT scanners from different manufacturers. Finally, the different acquisition modes (sequential/spiral and standard resolution/UHR) were not separately evaluated to determine whether these modes contributed to differences in image quality.

In conclusion, the new ZF reconstruction algorithm can improve image quality and diagnostic usability in photon-counting cCTA and can contribute to the evolving landscape, marking a step forward in the pursuit of non-invasive, artifact-free diagnostic imaging.

## Abbreviations

cCTA: Coronary CT angiography
SD: Standard reconstruction algorithm
TS: TrueStack reconstruction algorithm
UHR: Ultra-high-resolution
ZF: ZeeFree reconstruction algorithm
